# Barriers to Seeking Medical Consultation for Urinary Incontinence: A Nationwide Population‐Based Study in Saudi Arabia

**DOI:** 10.1111/luts.70033

**Published:** 2025-10-12

**Authors:** Ebtesam Almajed, Norah Alqntash, Badriyah AlDejain, Noura AlQurashi, Mohammed Alshehri, Ali AbdelRaheem, Nojoud Alamri

**Affiliations:** ^1^ Clinical Science Department, College of Medicine Princess Nourah Bint Abdulrahman University Riyadh Saudi Arabia; ^2^ College of Medicine Taif University Taif Saudi Arabia; ^3^ Department of Urology, King Abdullah Bin Abdulaziz University Hospital Princess Nourah Bint Abdulrahman University Riyadh Saudi Arabia; ^4^ Department of Urology Tanta University Hospital Tanta Egypt; ^5^ Department of Urology King Saud Medical City Riyadh Saudi Arabia

**Keywords:** barriers, cross‐sectional, medical consultation, Saudi Arabia, urinary incontinence

## Abstract

**Objectives:**

Urinary incontinence (UI) is prevalent and often underreported due to various barriers affecting healthcare‐seeking behavior. This study aimed to identify barriers preventing patients from seeking help for UI, assess the influence of sociodemographic and clinical factors on these barriers, and determine the associations between UI types and barriers in Saudi Arabia.

**Methods:**

A nationwide, cross‐sectional study was conducted from June 2024 to April 2025 among individuals aged ≥ 18 years who experienced UI and had not sought medical consultation. Participants completed a questionnaire that comprised sociodemographic data, the International Consultation on Incontinence Questionnaire‐Urinary Incontinence Short Form (ICIQ‐UI SF), and a modified Barriers to Incontinence Care Seeking Questionnaire (BICS‐Q). Data were analyzed using descriptive statistics, independent *t*‐tests, ANOVA, and binary logistic regression.

**Results:**

Of 505 eligible participants, 80.6% were female, predominantly aged 31–45 (40.0%), and the most common UI type was mixed UI (37.2%). The most significant barriers were embarrassment (33.3%), logistical inconvenience (appointments scheduled too far in advance, 36.8%), and provider‐related issues (lack of available providers, 12.3%). Gender, obesity, residency region, and type of UI significantly influenced barrier perception. Fear‐related barriers were notably higher in nocturnal UI, whereas embarrassment and cultural concerns were highest among those with mixed UI.

**Conclusion:**

This study highlights significant embarrassment, inconvenience, and provider‐related barriers deterring healthcare‐seeking among UI patients in Saudi Arabia. Findings emphasize the need for culturally tailored interventions, improved healthcare accessibility, and targeted public awareness campaigns.

## 1 Introduction

Urinary incontinence (UI) is one of the most prevalent yet concealed health issues across different populations [1]. The International Continence Society (ICS) defined UI as a “complaint of involuntary loss of urine” [2]. UI is widely recognized to adversely affect well‐being and quality of life [3, 4]. Globally, UI affects an estimated 423 million individuals aged 20 years and older [1], which suggests a pattern of underreporting that may lead to adverse consequences of urinary incontinence, including but not limited to shame, insecurity, social isolation, and depression [5].

Vasconcelos et al. conducted a systematic review identifying women's knowledge, attitude, and practices regarding UI. According to this review, insufficient knowledge about UI has been cited as one of the reasons for not seeking medical consultation, as many were unaware of treatment availability. A significant barrier to seeking care was shame and embarrassment, primarily when consulting with a male provider. Additional barriers included fear of surgery, lack of time, cost, inconvenience, not considering the symptoms severe enough to merit treatment, and limited access to healthcare services [6].

Locally, despite the high prevalence of UI, a study in 2020 in Saudi Arabia focused explicitly on determining reasons for not seeking treatment regarding UI in Taif City. They reported that out of 631 participants, only 21.7% sought medical care for UI, whereas 33.9% did not, with reasons including shame of seeking assistance, the belief that UI is a normal part of aging (18.7%), refusing to see a male physician (13.2%), and the belief that UI treatment did not exist (12.5%) [7]. Another 2020 study conducted in Saudi Arabia among women attending Ministry of Health Primary Healthcare Centers (PHCCs) in Riyadh reported that 42.6% of the 340 participants experienced urinary incontinence, yet 93.5% of those affected did not seek medical consultation. Furthermore, when asked about their perceptions of urinary incontinence, 41.8% of the women considered it a regular part of aging, and 52.6% were unaware that treatment options were available [8]. On the international level, among women residing in nursing homes, 77% experience urinary incontinence; however, only 25% of them seek help [9]. Although this statistic pertains specifically to institutionalized women, who are presumed to have greater access to healthcare services, it is plausible that a significant portion of the broader population similarly refrains from seeking assistance.

A thorough review of the literature and considering the unique characteristics of each country's social and cultural environment, there is a scarcity of information about barriers to seeking consultation for UI in Saudi Arabia. Identifying the predictors and reasons for help‐seeking behavior is an essential first step toward implementing effective health programs and policies. Therefore, this study was conducted to identify the barriers to seeking consultation for UI in Saudi Arabia.

## 2 Methods

### 2.1 Study Design and Population

This was a nationwide, cross‐sectional study conducted in Saudi Arabia between June 2024 and April 2025 using a convenience non‐probability sampling technique. Surveys were distributed to the patients attending the outpatient department (OPD) waiting areas, including Family Medicine, Obstetrics and Gynecology, Internal Medicine, Surgery, Orthopedics, and Pediatrics, at a single secondary institution in Saudi Arabia. Furthermore, the survey was distributed nationwide via social media platforms (X, WhatsApp, Telegram) to enhance reach and representativeness. Adults > 18 years or older residing in Saudi Arabia with UI who had not sought medical consultation and agreed to participate were eligible for inclusion. Participants were excluded if they were under 18, had already sought care for UI, did not complete the survey, or declined participation. Based on a 95% confidence level, 5% margin of error, and 80% power, the required sample size was calculated to be 376; however, more responses were collected to overcome potential incomplete or invalid entries.

### 2.2 Ethics Statement and Informed Consent

The study was reviewed and approved by the Institutional Review Board of Princess Nourah bint Abdulrahman University, Riyadh, Saudi Arabia (IRB Log Number: 23‐0277). The study was granted exempt status. Participation was voluntary and anonymous. Electronic informed consent was obtained at the beginning of the survey, and participants could exit at any point without obligation.

### 2.3 Study Survey and Data Collection

Data were collected electronically using Google Forms, and the questionnaire was available in Arabic and English. The survey consisted of four parts: (1) sociodemographic and gynecological data, including age, gender, marital and educational status, occupation, household income, medical and surgical history, smoking status, history of vaginal or abdominal gynecologic surgery, gravidity and parity, mode of delivery, menstrual and menopausal status, in addition to the number of physician visits per year; (2) the International Consultation on Incontinence Questionnaire‐Urinary Incontinence Short Form (ICIQ‐UI SF) [10], a validated 4‐item scale assessing the frequency, severity, and impact of UI on quality of life (score range: 0–21). The Arabic version of ICIQ‐UI SF was utilized, which is valid, reliable, and responsive; and (3) the modified Barriers to Incontinence Care Seeking Questionnaire (BICS‐Q) [11], developed by Heit et al. (2008). It demonstrated factor validity and reliability, consisting of 23 items on a 4‐point Likert scale addressing perceived barriers across five subscales, for example, inconvenience, relationships, cost, and site‐related factors. Originally, BICS‐Q consisted of 14 items; we added nine items based on previous studies that have identified barriers to seeking medical consultation and serve the objectives of our study. The respondents describe barriers to care as ‘not at all,’ ‘slightly,’ ‘moderately,’ or ‘greatly,’ with higher scores indicating a greater barrier to seeking incontinence care. The BICS‐Q was translated into Arabic and back‐translated to ensure conceptual equivalence, with clinical experts reviewing the final version.

### 2.4 Statistical Analysis and Data Management

A comprehensive statistical analysis was conducted on the dataset, encompassing descriptive and inferential methodologies. A descriptive analysis is conducted to summarize the participants' demographic characteristics, including age, gender, and other features. Moreover, independent *T*‐tests and ANOVA tests were used to assess the association between continuous variables. Subsequently, binary logistic regression was utilized to find the adjusted predictors for UI types and medical avoidance behavior. All statistical analyses are executed using IBM's SPSS Software, version 29.0.0.

## 3 Results

**FIGURE 1:**
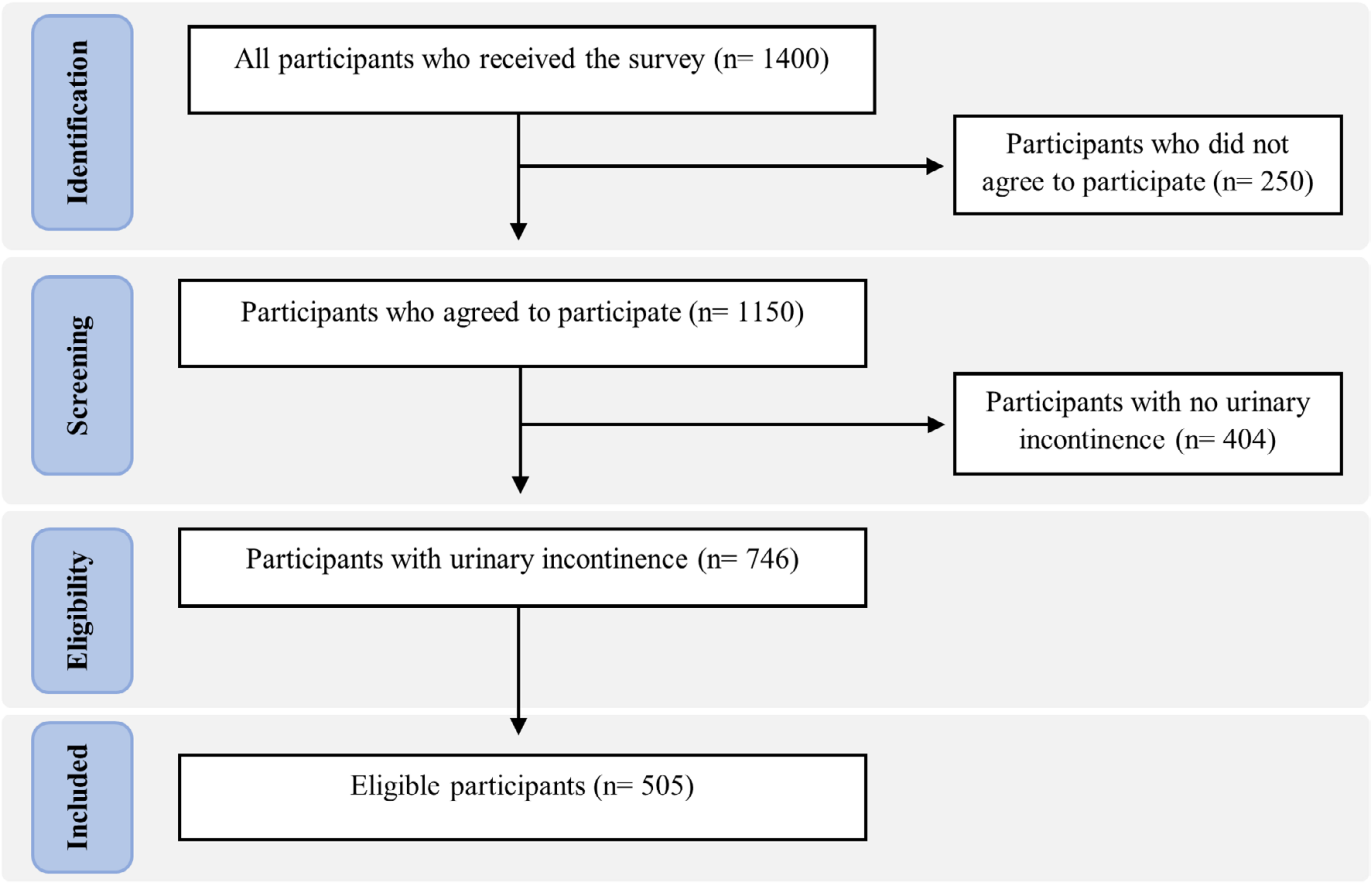
Flowchart of the study sample.

**FIGURE 2:**
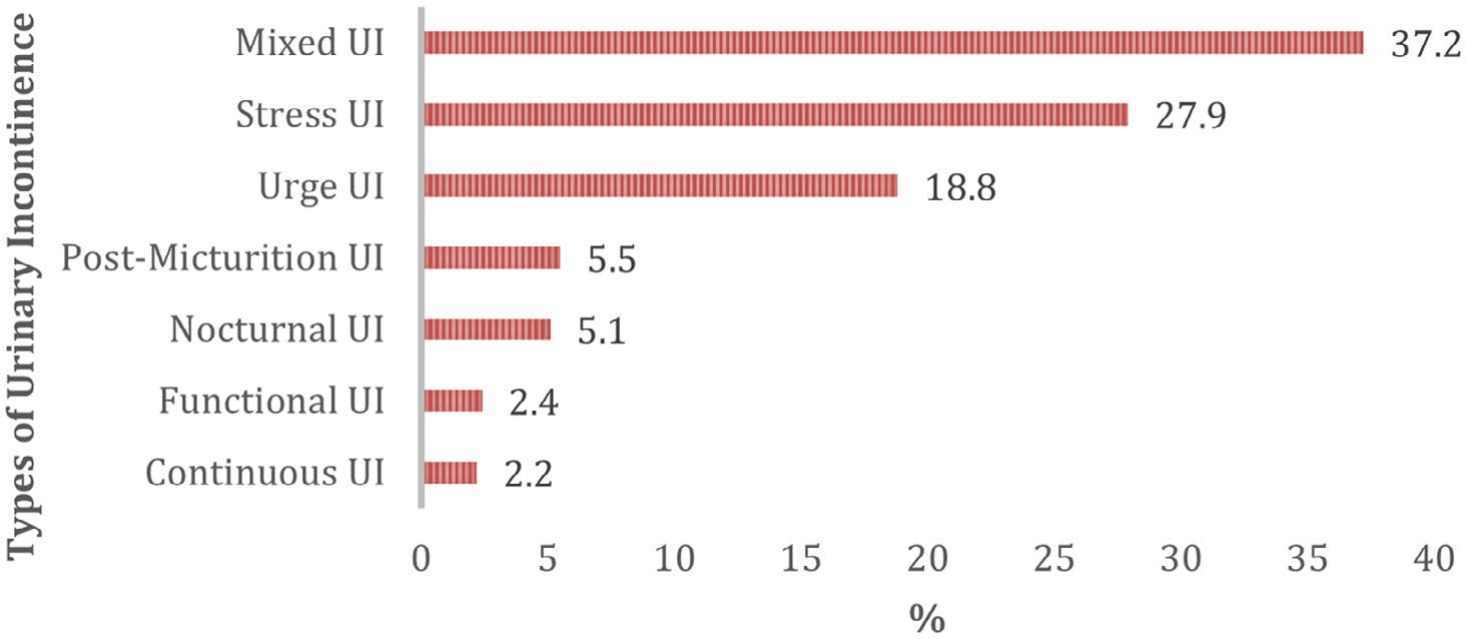
Types of urinary incontinence among the studied sample.

This study included 505 participants for the final analysis of the assessment of barriers against seeking consultation for urinary incontinence; Figure 1 displays the flowchart of the study sample. Most participants were female (80.6%) and Saudi nationals (92.1%). The largest age group was 31–45 years (40.0%), followed by 18–30 years (32.5%). In terms of BMI, normal weight was the most common category (33.7%), followed by overweight (30.5%). The central region had the highest representation (*n* = 255, 50.5%). The majority of participants were married (59.0%) and held a bachelor's degree (64.8%). Regarding employment, 30.1% were employed and 26.7% were housewives. Nearly half of the participants (49.9%) reported a monthly income below 5000 SAR. Most were non‐smokers (71.7%), and 52.1% reported having chronic diseases, with thyroid disorders being the most prevalent (36.9%). A majority reported 1–4 doctor visits in the past year (51.7%). Among female participants, 41.5% had a history of gynecologic surgery, and the average number of pregnancies was 3.2 ± 2.8. Vaginal delivery was the most common mode of birth (51.0%), and 57.7% reported having a regular menstrual cycle. Regarding urinary incontinence, 39.2% of participants reported experiencing urine leakage about once a week (*n* = 198), followed by two or three times a week (25.1%, *n* = 127). Daily leakage was reported by 15.8% (*n* = 80), while 15.6% (*n* = 79) experienced leakage several times a day, and 4.2% (*n* = 21) reported constant leakage. Most participants described the amount of urine lost as small (71.9%, *n* = 363), followed by moderate (22.6%, *n* = 114), and large (5.5%, *n* = 28). The mean interference score of urine leakage in daily life, measured on a 0–10 scale, was 4.1 ± 3.1, indicating a mild to moderate impact on daily functioning. Regarding the type of urinary incontinence, mixed UI was the most commonly reported type (37.2%), as shown in Figure 2. These sociodemographic and clinical characteristics are summarized in Table 1.

**TABLE 1:** Sociodemographic and clinical characteristics of urinary incontinence of the participants (*n* = 505).

	Response	Frequency *N* (%)
Gender	Female	407 (80.6)
	Male	98 (19.4)
Age	18–30 years	164 (32.5)
	31–45 years	202 (40.0)
	46–60 years	108 (21.4)
	> 60 years	31 (6.1)
BMI	Underweight	36 (7.1)
	Normal	170 (33.7)
	Overweight	154 (30.5)
	Obese class 1	91 (18.0)
	Obese class 2	32 (6.3)
	Obese class 3	22 (4.4)
Nationality	Non‐Saudi	40 (7.9)
	Saudi	465 (92.1)
Residency	Central	255 (50.5)
	Western	106 (21.0)
	Eastern	77 (15.2)
	Southern	37 (7.3)
	Northern	30 (5.9)
Marital status	Single	147 (29.1)
	Married	298 (59.0)
	Divorced	43 (8.5)
	Widowed	17 (3.4)
Highest educational level	School	128 (25.3)
	Bachelor's	327 (64.8)
	Postgraduate	50 (9.9)
Occupation status	Unemployed	77 (15.2)
	Employee	152 (30.1)
	Housewife	135 (26.7)
	Students	91 (18.0)
	Retired	50 (9.9)
Average monthly income	< 5000 SAR	252 (49.9)
	5000–10 000 SAR	152 (30.1)
	> 10 000 SAR	101 (20.0)
Smoking status	Non‐smoker	362 (71.7)
	Current smoker	61 (12.1)
	Passive smoker	75 (14.9)
	Previous smokers	7 (1.4)
Chronic diseases	No	242 (47.9)
	Yes	263 (52.1)
Type of disease	Thyroid disease	97 (36.9)
	Diabetes	74 (28.1)
	Hypertension	74 (28.1)
	Chronic constipation	60 (22.8)
	Asthma	55 (20.9)
	Cardiovascular disease	31 (11.8)
	Others	22 (8.4)
Doctor visits (past year)	None	117 (23.2)
	1–4 visits	261 (51.7)
	> 5 visits	127 (25.1)
Obstetrics and gynecological history		
Vaginal or abdominal gynecologic surgery history	No	238 (58.5)
	Yes	169 (41.5)
No. of times get pregnant	Mean (SD)	3.2 (2.8)
	Median	3
No. of times pregnancy delivered	Mean (SD)	2.7 (2.3)
	Median	3
Mode of delivery	Never	115 (28.5)
	Vaginal delivery	206 (51.0)
	C‐section	30 (7.4)
	Both	53 (13.1)
Menstrual history	Regular period	235 (57.7)
	Irregular period	99 (24.3)
	Postmenopausal	73 (17.9)
Clinical characteristics of UI		
Urine leakage frequency	About once a week	198 (39.2)
	Two or three times a week	127 (25.1)
	About once a day	80 (15.8)
	Several times a day	79 (15.6)
	All the time	21 (4.2)
Urine leakage amount	Small amount	363 (71.9)
	Moderate amount	114 (22.6)
	Large amount	28 (5.5)
Interference of urine leakage on everyday life (on 0–10 scale)	Mean (SD)	4.1 (3.1)

The various barriers to seeking consultation for urinary incontinence, categorized by subdomains, are presented in Table 2. Among site‐related barriers, over half of respondents in both age groups reported no issues with transportation (≤ 45: 59.3%; > 45: 62.6%) or clinic distance (≤ 45: 50.0%; > 45: 48.9%). For the cost‐related barriers, the most common issue was the high cost of the diagnostic evaluation, with 11.7% of those aged ≤ 45 and 15.1% of those aged > 45 reporting this as a great barrier. Complexity and delays in insurance were less frequently reported as significant concerns. Provider‐related issues, such as lack of availability and inadequate explanation, were more prominent in older adults compared to younger ones. Fear‐related barriers were more significant among younger participants, particularly discomfort with examination (greatly: ≤ 45 = 15.0%, > 45 = 12.2%) and fear of diagnosis (≤ 45 = 14.2%). Notably, among the additional barriers, embarrassment, preference for same‐sex physicians, and lack of knowledge showed consistently higher impact in both age groups, especially > 45 years for same‐sex physician preference (38.1% greatly).

**TABLE 2:** Different barriers according to their subdomains against seeking consultation for urinary incontinence.

		*N* (%)			
		Not at all	Slightly	Moderately	Greatly
Site‐related barriers					
Lack of transportation to the clinic	< 45 years	217 (59.3%)	56 (15.3%)	62 (16.9%)	31 (8.5%)
	≥ 45 years	87 (62.6%)	30 (21.6%)	10 (7.2%)	12 (8.6%)
The clinic location is too distant	< 45 years	183 (50.0%)	78 (21.3%)	69 (18.9%)	36 (9.8%)
	≥ 45 years	68 (48.9%)	31 (22.3%)	29 (20.9%)	11 (7.9%)
Cost‐related barriers					
Delayed insurance reimbursement	< 45 years	236 (64.5%)	56 (15.3%)	37 (10.1%)	37 (10.1%)
	≥ 45 years	86 (61.9%)	21 (15.1%)	14 (10.1%)	18 (12.9%)
The insurance process is too complex	< 45 years	230 (62.8%)	68 (18.6%)	42 (11.5%)	26 (7.1%)
	≥ 45 years	88 (63.3%)	23 (16.5%)	12 (8.6%)	16 (11.5%)
High cost of diagnostic evaluation	< 45 years	204 (55.7%)	65 (17.8%)	54 (14.8%)	43 (11.7%)
	≥ 45 years	78 (56.1%)	19 (13.7%)	21 (15.1%)	21 (15.1%)
Provider related barriers					
Lack of available healthcare providers	< 45 years	157 (42.9%)	92 (25.1%)	76 (20.8%)	41 (11.2%)
	≥ 45 years	43 (30.9%)	43 (30.9%)	32 (23.0%)	21 (15.1%)
Inadequate explanation by the provider	< 45 years	197 (53.8%)	66 (18.0%)	66 (18.0%)	37 (10.1%)
	≥ 45 years	71 (51.1%)	29 (20.9%)	23 (16.5%)	16 (11.5%)
Perceived disinterest from the provider/staff	< 45 years	211 (57.7%)	84 (23.0%)	39 (10.7%)	32 (8.7%)
	≥ 45 years	79 (56.8%)	36 (25.9%)	22 (15.8%)	2 (1.4%)
Inconvenience‐related barriers					
Excessive waiting time at appointments	< 45 years	214 (58.5%)	75 (20.5%)	51 (13.9%)	26 (7.1%)
	≥ 45 years	86 (61.9%)	37 (26.6%)	11 (7.9%)	5 (3.6%)
Appointments are scheduled too far in advance	< 45 years	189 (51.6%)	67 (18.3%)	55 (15.0%)	55 (15.0%)
	≥ 45 years	70 (50.4%)	32 (23.0%)	20 (14.4%)	17 (12.2%)
Limited office hours	< 45 years	170 (46.4%)	76 (20.8%)	68 (18.6%)	52 (14.2%)
	≥ 45 years	78 (56.1%)	24 (17.3%)	29 (20.9%)	8 (5.8%)
Fear‐related barriers					
Fear of healthcare providers	< 45 years	214 (58.5%)	75 (20.5%)	51 (13.9%)	26 (7.1%)
	≥ 45 years	86 (61.9%)	37 (26.6%)	11 (7.9%)	5 (3.6%)
Discomfort with being examined/questioned	< 45 years	189 (51.6%)	67 (18.3%)	55 (15.0%)	55 (15.0%)
	≥ 45 years	70 (50.4%)	32 (23.0%)	20 (14.4%)	17 (12.2%)
Fear of receiving a serious diagnosis	< 45 years	170 (46.4%)	76 (20.8%)	68 (18.6%)	52 (14.2%)
	≥ 45 years	78 (56.1%)	24 (17.3%)	29 (20.9%)	8 (5.8%)
Additional barriers					
Belief that urinary incontinence is a normal part of aging	< 45 years	192 (52.5%)	92 (25.1%)	56 (15.3%)	26 (7.1%)
	≥ 45 years	53 (38.1%)	40 (28.8%)	31 (22.3%)	15 (10.8%)
Perceived lack of effective treatment for UI	< 45 years	177 (48.4%)	77 (21.0%)	74 (20.2%)	38 (10.4%)
	≥ 45 years	64 (46.0%)	36 (25.9%)	26 (18.7%)	13 (9.4%)
Embarrassment discussing UI	< 45 years	132 (36.1%)	83 (22.7%)	79 (21.6%)	72 (19.7%)
	≥ 45 years	54 (38.8%)	41 (29.5%)	20 (14.4%)	24 (17.3%)
Embarrassment during physical examination	< 45 years	121 (33.1%)	87 (23.8%)	67 (18.3%)	91 (24.9%)
	≥ 45 years	41 (29.5%)	41 (29.5%)	21 (15.1%)	36 (25.9%)
Preference for a same‐sex physician	< 45 years	102 (27.9%)	86 (23.5%)	63 (17.2%)	115 (31.4%)
	≥ 45 years	37 (26.6%)	25 (18.0%)	24 (17.3%)	53 (38.1%)
Lack of knowledge about the appropriate specialist	< 45 years	156 (43.1%)	66 (18.2%)	76 (21.0%)	64 (17.7%)
	≥ 45 years	56 (40.6%)	40 (29.0%)	22 (15.9%)	20 (14.5%)
Presence of more pressing health concerns	< 45 years	163 (44.5%)	86 (23.5%)	62 (16.9%)	55 (15.0%)
	≥ 45 years	58 (41.7%)	25 (18.0%)	35 (25.2%)	21 (15.1%)
Self‐management is perceived as sufficient	< 45 years	183 (50.0%)	90 (24.6%)	61 (16.7%)	32 (8.7%)
	≥ 45 years	63 (45.3%)	29 (20.9%)	30 (21.6%)	17 (12.2%)
Lack of support or discouragement from others	< 45 years	239 (65.3%)	62 (16.9%)	46 (12.6%)	19 (5.2%)
	≥ 45 years	70 (50.4%)	32 (23.0%)	20 (14.4%)	17 (12.2%)

Table 3 shows the impact of various barrier categories on the consultation‐seeking behavior for urinary incontinence. Among participants aged ≤ 45 years, the highest mean score was observed for the additional barriers (9.38 ± 6.05), which contributed 34.74% of the total possible score, followed by inconvenience‐related barriers (2.86 ± 2.72, 31.78%) and fear‐related barriers (2.64 ± 2.62, 29.33%). The provider‐related barriers also had a notable impact (2.55 ± 2.45, 28.33%), whereas site‐related (1.63 ± 1.85, 27.17%) and cost‐related barriers (2.11 ± 2.52, 23.44%) contributed less. For participants over 45 years, the additional barriers again had the highest influence (10.20 ± 5.40, 37.78%), followed by provider‐related barriers (2.73 ± 2.22, 30.33%) and inconvenience‐related barriers (2.83 ± 2.60, 31.44%). The fear‐related barriers (2.18 ± 2.26, 24.22%) were less influential in this group, while the cost‐related barriers (2.32 ± 2.86, 25.78%) and site‐related barriers (1.50 ± 1.69, 25.00%) remained the most influential. Overall, the total barrier score was similar between the age groups, with 21.18 ± 14.25 (30.68%) in ≤ 45 years and 21.78 ± 12.17 (31.55%) in > 45 years.

**TABLE 3:** Summary statistics of barrier categories impacting consultation‐seeking behavior for urinary incontinence, categorized by age.

	≤ 45 years		> 45 years		Range (min–max)
	Mean (SD)	Score %	Mean (SD)	Score %	
Site‐related barriers	1.63 (1.85)	27.17%	1.50 (1.69)	25.00%	0–6
Cost‐related barriers	2.11 (2.52)	23.44%	2.32 (2.86)	25.78%	0–9
Provider‐related barriers	2.55 (2.45)	28.33%	2.73 (2.22)	30.33%	0–9
Inconvenience‐related barriers	2.86 (2.72)	31.78%	2.83 (2.60)	31.44%	0–9
Fear‐related barriers	2.64 (2.62)	29.33%	2.18 (2.26)	24.22%	0–9
Additional barriers	9.38 (6.05)	34.74%	10.20 (5.40)	37.78%	0–27
Total barrier score	21.18 (14.25)	30.68%	21.78 (12.17)	31.55%	0–69

Table 4 demonstrates the association between different types of UI and the barriers to seeking medical consultation for UI. Notably, for the fear‐related barriers, the highest scores were reported by participants with nocturnal UI (4.50 ± 2.42) (*p* = 0.005). In contrast, those with continuous UI reported the lowest fear barrier scores (1.73 ± 1.68). Additional barriers were likewise significantly different across the UI types (*p* = 0.005), with the highest scores in nocturnal UI (12.04 ± 5.09) and mixed UI (10.29 ± 5.98), while the lowest was seen in functional UI (7.33 ± 7.05).

**TABLE 4:** Association of different UI types with barriers in seeking medical consultancy for UI.

	Mean (SD)							*p* ^a^
	Mixed UI	Stress UI	Urge UI	Post‐ micturition UI	Nocturnal UI	Functional UI	Continuous UI	
Site barriers	1.66 (1.85)	1.51 (1.83)	1.46 (1.74)	1.29 (1.82)	2.42 (1.63)	1.42 (1.38)	1.09 (1.30)	0.209
Cost barriers	2.18 (2.77)	2.31 (2.57)	1.86 (2.41)	1.71 (2.46)	2.88 (2.52)	1.67 (2.31)	1.73 (2.10)	0.505
Relationship barriers	3.00 (2.65)	2.22 (2.17)	2.22 (1.98)	2.82 (2.92)	2.77 (2.03)	2.58 (2.02)	2.45 (1.97)	0.067
Inconvenience barriers	3.11 (2.77)	2.56 (2.71)	2.56 (2.38)	3.11 (3.26)	2.96 (2.31)	2.67 (2.46)	3.73 (2.69)	0.423
Fear barriers	2.35 (2.75)	2.49 (2.45)	2.36 (1.98)	2.57 (2.75)	4.50 (2.42)	2.17 (2.92)	1.73 (1.68)	***0.005***
Additional barriers	10.29 (5.98)	8.17 (5.72)	10.09 (5.35)	8.96 (6.69)	12.04 (5.09)	7.33 (7.05)	9.09 (5.38)	***0.005***
Combined all barrier	22.62 (14.09)	19.26 (14.54)	20.53 (10.91)	20.59 (16.18)	27.58 (11.63)	17.83 (12.88)	19.82 (7.11)	0.068

*Note:* Bold and italic value indicates *P* < 0.05.

^a^ ANOVA.

Table 5 presents the adjusted odds ratios (AORs) for non‐consultation among women reporting bothersome UI, stratified by UI subtype. Among participants with mixed UI, being male was significantly associated with lower odds of avoiding medical consultation (AOR = 0.17, *p* < 0.05), while higher body mass index (BMI) (AOR = 1.05, *p* < 0.05) and the presence of chronic diseases (AOR = 1.56, *p* < 0.05) were independently associated with increased likelihood of non‐consultation. In contrast, elevated BMI was a protective factor for stress UI, significantly decreasing the odds of avoiding consultation (AOR = 0.93, *p* < 0.05). Among individuals with urge UI, increasing age was associated with higher odds of non‐consultation (AOR = 1.03, *p* < 0.05), whereas a higher monthly income was found to be protective (AOR = 0.68, *p* < 0.05). For participants classified under “other UI” types, being male markedly increased the likelihood of not seeking medical care (AOR = 5.57, *p* < 0.05), while younger age (AOR = 0.96, *p* < 0.05) and the presence of chronic illness (AOR = 0.36, *p* < 0.05) were significant protective factors.

**TABLE 5:** Association between socioeconomic status with non‐consultation behavior among women experiencing bothersome urinary incontinence.

	Mixed UI	Stress UI	Urge UI	Other UI
	AOR (95% CI)	AOR (95% CI)	AOR (95% CI)	AOR (95% CI)
Gender (male)	**0.17 (0.08–0.34)** *	1.57 (0.91–2.71)	0.73 (0.38–1.44)	**5.57 (2.94–10.55)** *
Age	0.99 (0.97–1.01)	1.00 (0.98–1.02)	**1.03 (1.01–1.06)** *	**0.96 (0.94–0.99)** *
BMI	**1.05 (1.01–1.08)** *	**0.93 (0.89–0.96)** *	1.00 (0.96–1.04)	1.02 (0.97–1.06)
Nationality (Saudi)	0.49 (0.24–1.02)	2.19 (0.92–5.24)	1.56 (0.62–3.97)	0.91 (0.32–2.58)
Marital status (divorced/widow)	1.39 (0.98–1.96)	0.80 (0.55–1.16)	0.69 (0.46–1.04)	1.28 (0.79–2.08)
Higher educational	1.09 (0.74–1.61)	1.29 (0.86–1.94)	0.71 (0.44–1.13)	1.07 (0.63–1.82)
Unemployed	1	1	1	1
Employee	1.30 (0.65–2.59)	0.52 (0.26–1.04)	1.94 (0.86–4.38)	1.03 (0.40–2.71)
Housewife	1.25 (0.64–2.43)	1.00 (0.51–1.95)	1.06 (0.47–2.38)	0.47 (0.15–1.50)
Students	0.95 (0.44–2.08)	0.67 (0.33–1.34)	0.66 (0.26–1.65)	2.57 (1.00–6.61)
Retired	1.40 (0.54–3.61)	0.58 (0.22–1.55)	0.86 (0.28–2.64)	2.11 (0.59–7.59)
Higher monthly income	1.26 (0.93–1.72)	0.74 (0.53–1.03)	**0.68 (0.46–0.99)** *	1.48 (0.95–2.29)
Smoker (yes)	1.25 (0.97–1.60)	0.90 (0.68–1.18)	0.78 (0.57–1.08)	1.06 (0.75–1.50)
Chronic diseases (yes)	**1.56 (1.02–2.40)** *	0.91 (0.58–1.43)	1.20 (0.72–2.00)	**0.36 (0.19–0.68)** *

*Note:* Bold value indicates *P* < 0.05.

* Shows *p* < 0.05 and significant.

The associations between various sociodemographic and clinical characteristics and the different domains of barriers to seeking medical consultation for UI are shown in Tables S1 and S2. For site‐related barriers, statistically significant associations were identified with BMI (Obese Class III: *M* = 2.50, SD = 2.11; *p* = 0.036), marital status (widowed individuals: *M* = 2.76, SD = 1.95; *p* = 0.049), region of residency (southern region; *p* = 0.028), monthly income (5000–10 000 SAR; *p* = 0.011), history of gynecologic surgery (*p* = 0.044), and irregular menstrual cycles (*p* = 0.047).

For cost‐related barriers, significant associations were observed with gender (males: *M* = 2.92, SD = 2.44 vs. females: *M* = 1.99, SD = 2.63; *p* = 0.002), BMI (Obese Class III: *M* = 4.05, SD = 3.84; *p* < 0.001), smoking status (*p* = 0.022), history of gynecologic surgery (*p* = 0.004), and C‐section delivery (*p* = 0.009). Furthermore, provider‐related barriers were significantly associated with BMI (Class III: *M* = 4.41, SD = 2.54; *p* < 0.001), region of residency (*p* = 0.025), income (5000–10 000 SAR; *p* = 0.027), gynecologic surgery (*p* = 0.005), and mode of delivery (*p* = 0.017). As for the inconvenience‐related barriers, it showed significant associations with BMI (Class III: *M* = 4.55, SD = 3.32; *p* < 0.001), region of residency (*p* = 0.039), income (*p* = 0.043), and mode of delivery (*p* = 0.039). Additional barriers which included embarrassment, privacy, and knowledge factors were significantly higher among participants with Class III obesity (*M* = 11.27, SD = 5.75; *p* = 0.032), those residing in the southern region (*M* = 11.19, SD = 5.14; *p* = 0.046), passive smokers (*M* = 11.64, SD = 5.52; *p* = 0.010), individuals with chronic diseases (*M* = 10.16, SD = 5.43; *p* = 0.027), and those with a history of irregular menstruation (*M* = 10.88, SD = 5.59; *p* = 0.011).

## 4 Discussion

UI is a prevalent condition that significantly impacts individuals' quality of life but remains underdiagnosed and undertreated in many settings [12]. Prior local research has shown that although UI affects a substantial proportion of Saudi women, only a minority seek medical help, often because they do not perceive it as a serious health problem [7, 12]. In Saudi Arabia, the issue is compounded by cultural factors, for example, stigma and modesty concerns that further complicate the landscape of healthcare‐seeking behavior [13]. Such findings underscored the need for a comprehensive nationwide assessment of why patients avoid consultation for UI. Our study was motivated by this gap to investigate the specific barriers preventing patients from seeking care for UI and to understand how sociocultural factors might shape these barriers. By focusing on a Saudi context, we aimed to identify actionable insights relevant to the Kingdom's healthcare system and cultural norms.

Saudi Arabia's healthcare system is a hybrid of public and private models. The public sector, led by the Ministry of Health and other government agencies such as the Armed Forces and the Ministry of Interior, provides free healthcare services to Saudi citizens and selected expatriates, covering primary care, hospital services, medications, and specialized treatments. In contrast, the private healthcare sector is regulated under the Cooperative Health Insurance System (CHIS). Private insurance options offer tiered plans (e.g., Bronze, Silver, Gold) with varying coverage levels and access to different providers. These plans cover a wide range of services, including outpatient visits, hospitalization, medications, surgeries, maternity care, and emergency services. Under CHIS, most insured patients do not pay upfront for medical services. Instead, the system operates through a direct billing model between healthcare providers (such as hospitals and clinics) and insurance companies. Patients receive care, and the provider bills the insurer directly [14]. However, reimbursement from insurers can sometimes face delays and is one of the commonly cited challenges by private providers, especially when documentation is incomplete or when disputes arise over coverage.

In terms of workforce demographics, the field of urology in Saudi Arabia remains predominantly male, with studies showing that approximately 98.8% of practicing urologists are men [15]. This disparity highlights the need for continued efforts to support and encourage female participation in surgical subspecialties.

Our nationwide cross‐sectional study identified personal/cultural etiologies, which constituted the largest share of the total barrier score, and inconvenience‐related barriers as predominant. Embarrassment and preferences related to provider gender emerged as leading concerns, highlighting the deep social stigma attached to UI. Additionally, logistical issues and fear‐related concerns significantly deterred seeking consultation. Men exhibited higher fear‐based barriers, while women particularly emphasized embarrassment and the necessity of a female urologist or a female urogynecologist. Body mass index (BMI) was another influential factor. Regionally, respondents from Southern Saudi Arabia faced more significant barriers, potentially reflecting disparities in healthcare access and cultural conservatism.

Additionally, 16%–17% of individuals were unsure which doctor to consult for UI, reflecting a knowledge gap that can delay or prevent treatment. Collectively, these patterns indicate that barriers to seeking care for UI are not uniform across the population; they are shaped by a person's social context and health background. Furthermore, our study examined how the type of UI and symptom characteristics relate to consultation avoidance. Interestingly, most barrier domains did not significantly differ between UI subtypes except for fear‐related and personal/cultural barriers. Higher fear‐related barriers, whether fear of the diagnostic process, potential underlying condition, or perhaps being judged, were evident among nocturnal UI patients. Meanwhile, for the social/cultural barriers, nocturnal UI again yielded the highest scores, alongside mixed UI, whereas those with functional UI reported the least social inhibition or embarrassment barriers.

Help‐seeking rates in our study are broadly consistent with the international literature on low medical consultation for UI. Research from various countries has repeatedly shown that well under half of those suffering from incontinence ever reach out for professional help. For instance, analysis from the long‐running Nurses' Health Study in the United States found that only about one‐third of middle‐aged and older women with UI had ever discussed their symptoms with a clinician [16]. Similarly, European population studies reported low consultation rates; in Denmark, only 29% of women with UI sought care [17]. Moreover, a mere 17% of women with UI in the United Kingdom saw a healthcare provider about it [18]. Although our study population and methods differ, the implication is that Saudi Arabia faces a comparable challenge, potentially even more pronounced, given the cultural factors needed to close the gap between UI prevalence and treatment rates.

Our findings align with and add new dimensions to the literature on help‐seeking behavior in urinary incontinence. Embarrassment, stigma, and normalization of symptoms emerged as central themes in our study, which align with several studies from other regions [19, 20]. A recent systematic review encompassing over 20 000 women across high‐income countries identified stigma and lack of knowledge as barriers to care for pelvic floor disorders, including UI [21]. Women commonly feel ashamed of incontinence and may internalize the belief that it is a normal part of aging or childbirth that they must tolerate rather than a medical condition deserving treatment [21]. This is vividly reflected in a local Saudi study, where nearly three‐quarters of women with UI did not seek help, and the predominant rationale for non‐treatment was the perception that UI did not warrant medical attention, followed by feelings of embarrassment about discussing it [12]. Our nationwide results echo these observations. Furthermore, a potential strategy to overcome embarrassment and inconvenience barriers identified in our study, as emphasized by Zhao et al. [22] is that eHealth interventions might reduce embarrassment by providing a discreet alternative to face‐to‐face consultations.

We similarly found evidence of knowledge gaps about uncertainty about available treatments or whom to consult, which is in accordance with findings from both Middle Eastern and Western contexts where patients often do not realize that effective UI therapies exist or assume that nothing can be done [11, 20]. Moreover, our study highlights cultural and systemic differences that distinguish the Saudi context from some Western settings. One such difference is the importance of gender‐concordant care. The desire for a same‐sex physician, which over 30% of our participants cited as a greatly impacting barrier, is a relatively prominent issue in Saudi Arabia. In more liberal healthcare environments, while patients may have preferences for a provider of a specific gender, it is seldom reported as a reason to avoid care, especially to the extent observed here entirely. In our sample, which is predominantly female participants, many would rather delay or forego treatment than discuss incontinence with a male doctor. A prior Saudi survey noted this concern on a smaller scale. About 4% of women there expressed unwillingness to see a male doctor for UI [12]. However, our results suggest the issue is more widespread when considering the general population, possibly reflecting that in community settings, women who are uncomfortable with available male providers are simply never present at all.

According to our findings, obesity was associated with higher perceived barriers, especially regarding convenience and healthcare provider‐related barriers. Obesity is prevalent in Saudi Arabia and contributes to high UI rates [23]. Nevertheless, paradoxically, being obese in our sample correlated with more difficulty seeking care. Some international studies have implied similar patterns: Women with higher BMI often endure UI longer before seeking help, sometimes due to past negative healthcare encounters or because they feel ashamed to address a condition that they may attribute to their lifestyle [24]. Healthcare provider attitudes and knowledge significantly influence UI management. Vuuren et al. [25] concluded that inadequate knowledge and discomfort among healthcare providers highlight the need for provider education to improve patient comfort and consultation rates. Similarly, Gerritsen et al. [26] indicated that patient decision aids could enhance shared decision‐making by addressing patient concerns proactively, potentially reducing fear and embarrassment.

This study has important implications for public health and clinical practice in Saudi Arabia as it offers insights into the barriers faced by patients with UI. Public education through initiatives aimed at normalizing UI as a medical condition requiring professional intervention addresses stigma and embarrassment. Furthermore, improved logistical arrangements with expanded clinic hours and telehealth by leveraging digital health technologies options could alleviate the top cited barriers in the studied sample. Future research should explore qualitative investigations into the personal experiences of patients with UI, assess the effectiveness of educational and eHealth interventions, and further evaluate gender‐specific interventions.

The study has several limitations that should be acknowledged. First, its cross‐sectional design limits the ability to infer causality between identified barriers and consultation‐seeking behaviors. Second, the use of a convenience sampling method, based on responses collected from outpatient clinics and social media platforms, may have introduced selection bias. This approach, coupled with the overrepresentation of participants from the central region, potentially limits the generalizability of the findings to the broader Saudi population and may have influenced the regional comparison of perceived barriers.

In conclusion, this nationwide cross‐sectional study highlights a multifaceted array of barriers that deter patients from seeking medical consultation for UI in Saudi Arabia. Predominantly, embarrassment, inconvenience, and provider‐related concerns significantly impede healthcare‐seeking behaviors. Sociodemographic factors, including obesity, gender, and geographic location, have influenced these barriers. Our findings underscore the necessity for culturally sensitive public health interventions, increased awareness about available treatments, and improved healthcare accessibility, particularly addressing preferences for gender‐concordant care providers. Future research should explore targeted strategies, including educational campaigns and e‐health interventions.

## Author Contributions

Conceptualization: E.M. and M.S.H. Methodology: E.M. and N.Q. Validation: E.M. Formal analysis: E.M. Investigation: E.M., N.Q. and K.H.M. Resources: E.M., N.Q. and B.D. Data curation: E.M., N.Q. and B.D. Writing – original draft preparation: E.M., N.Q., B.D., and N.Q. Writing – review and editing. All authors: supervision. M.S.H. and A.A.: Project administration: E.M. Funding acquisition: E.M. and M.S.H. All authors have read and agreed to the published version of the manuscript.

## Ethics Statement

The study received ethical approval from the Institutional Review Board (IRB) at King Abdullah bin Abdulaziz University Hospital (approval number: 23‐0277). All participants provided informed consent. Responses were collected anonymously, and no identifying personal information was stored.

## Consent

Informed consent was obtained from all subjects involved in the study.

## Conflicts of Interest

The authors declare no conflicts of interest.

## Supporting information


**Data S1:** luts70033‐sup‐0001‐Tables.docx.

## Data Availability

The data that support the findings of this study are available from the corresponding author upon reasonable request.
