# Supplementary material for: Barriers to Seeking Medical Consultation for Urinary Incontinence: A Nationwide Population‐Based Study in Saudi Arabia
Source: Low Urin Tract Symptoms. 2025 Oct 12;17(6):e70033. doi: 10.1111/luts.70033 (PMC12515558; doi:10.1111/luts.70033)
Supplement: Supplementary file 1 — Data S1: luts70033‐sup‐0001‐Tables.docx. [file LUTS-17-e70033-s001.docx]

| Supplementary Table 1. Association of Sociodemographic Characteristics with Barriers in Seeking Help for Urinary Incontinence. | | | | | | | | | |  |
| --- | --- | --- | --- | --- | --- | --- | --- | --- | --- | --- |
|  | | **Site Barrier** | | **Cost Barriers** | | **Provider Barriers** | | **Inconvenience Barriers** | | |
|  |  | **Mean (SD)** | ***p*** | **Mean (SD)** | ***p*** | **Mean (SD)** | ***p*** | **Mean (SD)** | ***p*** | |
| Gender | Female | 1.52 (1.79) | 0.063^a^ | 1.99 (2.63) | ***0.002**** ^a^ | 2.52 (2.41) | 0.141 ^a^ | 2.78 (2.73) | 0.242 ^a^ | |
|  | Male | 1.90 (1.82) |  | 2.92 (2.44) |  | 2.92 (2.29) |  | 3.13 (2.50) |  |  |
| Age | 18-30 Years | 1.47 (1.75) | 0.303 ^b^ | 1.76 (2.32) | 0.082 ^b^ | 2.37 (2.35) | 0.412 ^b^ | 2.73 (2.76) | 0.414 ^b^ | |
|  | 31-45 Years | 1.76 (1.91) |  | 2.40 (2.65) |  | 2.70 (2.53) |  | 2.96 (2.68) |  |  |
|  | 46-60 Years | 1.56 (1.70) |  | 2.41 (2.83) |  | 2.81 (2.33) |  | 3.01 (2.71) |  |  |
|  | >60 Years | 1.26 (1.65) |  | 2.00 (3.02) |  | 2.45 (1.79) |  | 2.19 (2.12) |  |  |
| BMI | Underweight | 1.25 (2.01) | ***0.036**** ^b^ | 1.03 (1.68) | ***<0.001**** ^b^ | 1.78 (2.13) | ***<0.001****  ^b^ | 1.53 (2.25) | ***<0.001****  ^b^ | |
|  | Normal | 1.82 (1.77) |  | 2.55 (2.68) |  | 2.97 (2.35) |  | 3.09 (2.56) |  |  |
|  | Overweight | 1.40 (1.72) |  | 1.96 (2.49) |  | 2.23 (2.28) |  | 2.79 (2.67) |  |  |
|  | Obese Class 1 | 1.45 (1.69) |  | 1.79 (2.22) |  | 2.56 (2.48) |  | 2.92 (2.79) |  |  |
|  | Obese Class 2 | 1.50 (2.03) |  | 2.19 (3.01) |  | 2.19 (2.16) |  | 1.94 (2.30) |  |  |
|  | Obese Class 3 | 2.50 (2.11) |  | 4.05 (3.84) |  | 4.41 (2.54) |  | 4.55 (3.32) |  |  |
| Nationality | Non-Saudi | 1.60 (1.91) | 0.983 ^a^ | 2.27 (2.96) | 0.789 ^a^ | 3.00 (3.10) | 0.270 ^a^ | 2.48 (3.00) | 0.361 ^a^ | |
|  | Saudi | 1.59 (1.80) |  | 2.16 (2.59) |  | 2.57 (2.32) |  | 2.88 (2.66) |  |  |
| Marital Status | Single | 1.62 (1.78) | ***0.049**** ^b^ | 2.07 (2.22) | 0.342 ^b^ | 2.44 (2.26) | 0.755 ^b^ | 2.54 (2.47) | 0.241 ^b^ | |
|  | Married | 1.51 (1.81) |  | 2.20 (2.82) |  | 2.69 (2.49) |  | 2.93 (2.77) |  |  |
|  | Divorced | 1.63 (1.68) |  | 1.86 (2.13) |  | 2.51 (2.21) |  | 2.93 (2.82) |  |  |
|  | Widowed | 2.76 (1.95) |  | 3.18 (3.26) |  | 2.71 (2.17) |  | 3.76 (2.51) |  |  |
| Current Residency | Central | 1.63 (1.82) | ***0.028**** ^b^ | 2.22 (2.73) | 0.307 ^b^ | 2.70 (2.39) | ***0.025**** ^b^ | 2.95 (2.67) | ***0.039**** ^b^ | |
|  | Western | 1.28 (1.77) |  | 1.74 (2.41) |  | 2.01 (2.24) |  | 2.18 (2.74) |  |  |
|  | Eastern | 1.44 (1.71) |  | 2.21 (2.66) |  | 2.56 (2.51) |  | 2.94 (2.77) |  |  |
|  | Southern | 2.35 (1.80) |  | 2.57 (2.34) |  | 3.19 (2.01) |  | 3.51 (2.24) |  |  |
|  | Northern | 1.83 (1.88) |  | 2.63 (2.58) |  | 3.20 (2.66) |  | 3.27 (2.64) |  |  |
| Education Level | School | 1.38 (1.82) | 0.242 ^b^ | 2.04 (2.60) | 0.805 ^b^ | 2.42 (2.33) | 0.546 ^b^ | 2.92 (3.06) | 0.882 ^b^ | |
|  | Bachelor's | 1.69 (1.79) |  | 2.20 (2.69) |  | 2.64 (2.47) |  | 2.84 (2.54) |  |  |
|  | Postgraduate | 1.50 (1.85) |  | 2.26 (2.22) |  | 2.82 (1.97) |  | 2.70 (2.62) |  |  |
| Occupation Status | Unemployed | 1.84 (1.84) | 0.624 ^b^ | 2.18 (2.63) | 0.439 ^b^ | 2.56 (2.36) | 0.287 ^b^ | 2.71 (2.68) | 0.533 ^b^ | |
|  | Employee | 1.64 (1.72) |  | 2.08 (2.34) |  | 2.93 (2.43) |  | 2.94 (2.51) |  |  |
|  | Housewife | 1.56 (1.95) |  | 2.30 (3.03) |  | 2.52 (2.63) |  | 3.07 (3.17) |  |  |
|  | Students | 1.47 (1.68) |  | 1.84 (2.13) |  | 2.44 (2.21) |  | 2.76 (2.47) |  |  |
|  | Retired | 1.40 (1.81) |  | 2.66 (3.02) |  | 2.18 (1.83) |  | 2.34 (2.12) |  |  |
| Income | <5000 SAR | 1.49 (1.84) | ***0.011**** ^b^ | 2.06 (2.68) | 0.073 ^b^ | 2.36 (2.47) | ***0.027**** ^b^ | 2.81 (2.91) | ***0.043**** ^b^ | |
|  | 5000-10,000 SAR | 1.95 (1.79) |  | 2.56 (2.49) |  | 3.01 (2.19) |  | 3.22 (2.39) |  |  |
|  | >10,000 SAR | 1.33 (1.66) |  | 1.86 (2.61) |  | 2.58 (2.39) |  | 2.37 (2.44) |  |  |
| Smoking Status | No | 1.48 (1.77) | 0.119 ^b^ | 1.99 (2.63) | ***0.022**** ^b^ | 2.42 (2.42) | 0.069 ^b^ | 2.70 (2.72) | 0.296 ^b^ | |
|  | Yes | 1.98 (1.89) |  | 3.02 (2.46) |  | 3.07 (2.37) |  | 3.26 (2.53) |  |  |
|  | Passive Smoker | 1.79 (1.80) |  | 2.20 (2.50) |  | 3.04 (2.20) |  | 3.19 (2.58) |  |  |
|  | Previous Smokers | 2.14 (2.19) |  | 3.43 (3.21) |  | 3.00 (1.83) |  | 3.00 (3.32) |  |  |
| Chronic Diseases | No | 1.53 (1.75) | 0.467 ^a^ | 2.05 (2.61) | 0.313 ^a^ | 2.60 (2.57) | 0.994 ^a^ | 2.83 (2.74) | 0.892 ^a^ | |
|  | Yes | 1.65 (1.85) |  | 2.28 (2.63) |  | 2.60 (2.21) |  | 2.86 (2.63) |  |  |
| Doctor Visits | None | 1.52 (1.78) | 0.153 ^b^ | 2.36 (2.72) | 0.641 ^b^ | 2.32 (2.35) | 0.333 ^b^ | 2.55 (2.69) | 0.380 ^b^ | |
|  | 1-4 Visits | 1.74 (1.79) |  | 2.14 (2.56) |  | 2.67 (2.45) |  | 2.95 (2.64) |  |  |
|  | >5 Visits | 1.37 (1.84) |  | 2.06 (2.66) |  | 2.72 (2.29) |  | 2.91 (2.78) |  |  |
| Gynecologic Surgery | No | 1.37 (1.67) | ***0.044**** ^a^ | 1.67 (2.36) | ***0.004**** ^a^ | 2.24 (2.25) | ***0.005**** ^a^ | 2.59 (2.64) | 0.094 ^a^ | |
|  | Yes | 1.73 (1.94) |  | 2.43 (2.93) |  | 2.92 (2.57) |  | 3.05 (2.83) |  |  |
| Mode of Delivery | Never | 1.49 (1.77) | 0.095 ^b^ | 1.51 (1.95) | ***0.009**** ^b^ | 2.03 (2.10) | ***0.017**** ^b^ | 2.19 (2.45) | ***0.039**** ^b^ | |
|  | Vaginal Delivery | 1.41 (1.74) |  | 2.00 (2.65) |  | 2.58 (2.46) |  | 2.99 (2.83) |  |  |
|  | C-Section | 2.27 (2.21) |  | 3.23 (3.67) |  | 3.10 (2.66) |  | 3.20 (3.27) |  |  |
|  | Both | 1.68 (1.77) |  | 2.36 (2.96) |  | 3.15 (2.50) |  | 3.17 (2.38) |  |  |
| Menstrual History | Regular Period | 1.43 (1.76) | ***0.047**** ^b^ | 1.85 (2.56) | 0.472 ^b^ | 2.40 (2.50) | 0.257 ^b^ | 2.71 (2.75) | 0.544 ^b^ | |
|  | Irregular Period | 1.90 (1.96) |  | 2.19 (2.53) |  | 2.87 (2.38) |  | 3.04 (2.70) |  |  |
|  | Postmenopausal | 1.30 (1.61) |  | 2.15 (2.98) |  | 2.45 (2.10) |  | 2.66 (2.71) |  |  |
| (a) Independent T-Test, (b) ANOVA, (*) Significant Values | | | | | | | | | | |

| Supplementary Table 2. Association of Sociodemographic Characteristics with Barriers in Seeking Help for Urinary Incontinence. | | | | | | | |
| --- | --- | --- | --- | --- | --- | --- | --- |
|  | | **Fear Barrier** | | **Additional Barriers** | | **Overall Barriers** | |
|  |  | **Mean (SD)** | ***p*** | **Mean (SD)** | ***p*** | **Mean (SD)** | ***p*** |
| Gender | Female | 2.30 (2.49) | 0.001^a^ | 9.51 (5.89) | 0.444 ^a^ | 20.63 (13.39) | ***0.017**** ^a^ |
|  | Male | 3.38 (2.54) |  | 10.02 (5.89) |  | 24.33 (14.60) |  |
| Age | 18-30 Years | 2.87 (2.79) | 0.125 ^b^ | 9.27 (6.22) | 0.470 ^b^ | 20.49 (13.93) | 0.603 ^b^ |
|  | 31-45 Years | 2.45 (2.47) |  | 9.47 (5.92) |  | 21.75 (14.52) |  |
|  | 46-60 Years | 2.16 (2.29) |  | 10.39 (5.56) |  | 22.38 (12.61) |  |
|  | >60 Years | 2.26 (2.19) |  | 9.55 (4.82) |  | 19.71 (10.45) |  |
| BMI | Underweight | 2.39 (2.91) | ***<0.001**** ^b^ | 7.00 (5.06) | ***0.032**** ^b^ | 14.97 (11.48) | ***<0.001****  ^b^ |
|  | Normal | 3.06 (2.41) |  | 10.21 (5.84) |  | 23.73 (13.45) |  |
|  | Overweight | 2.05 (2.52) |  | 9.09 (5.76) |  | 19.54 (13.00) |  |
|  | Obese Class 1 | 2.14 (2.49) |  | 9.88 (6.61) |  | 20.75 (15.02) |  |
|  | Obese Class 2 | 2.09 (2.04) |  | 9.97 (4.65) |  | 19.88 (12.11) |  |
|  | Obese Class 3 | 3.77 (2.81) |  | 11.27 (5.75) |  | 30.55 (13.57) |  |
| Nationality | Non-Saudi | 2.48 (2.84) | 0.926 ^a^ | 9.21 (5.41) | 0.655 ^a^ | 21.08 (15.17) | 0.898 ^a^ |
|  | Saudi | 2.51 (2.51) |  | 9.64 (5.93) |  | 21.37 (13.59) |  |
| Marital Status | Single | 3.22 (2.62) | ***<0.001**** ^b^ | 9.94 (5.92) | 0.596 ^b^ | 21.88 (13.01) | 0.494 ^b^ |
|  | Married | 2.27 (2.47) |  | 9.61 (6.00) |  | 21.22 (14.27) |  |
|  | Divorced | 1.65 (2.11) |  | 8.53 (5.52) |  | 19.12 (12.90) |  |
|  | Widowed | 2.82 (2.58) |  | 9.47 (4.43) |  | 24.71 (11.02) |  |
| Current Residency | Central | 2.24 (2.38) | ***<0.001**** ^b^ | 9.67 (5.99) | ***0.046**** ^b^ | 21.42 (13.48) | ***0.003**** ^b^ |
|  | Western | 2.16 (2.37) |  | 8.25 (5.60) |  | 17.60 (12.83) |  |
|  | Eastern | 2.99 (2.79) |  | 10.13 (5.79) |  | 22.33 (14.42) |  |
|  | Southern | 3.89 (2.39) |  | 11.19 (5.14) |  | 26.70 (11.34) |  |
|  | Northern | 3.17 (3.06) |  | 10.57 (6.46) |  | 24.67 (16.35) |  |
| Education Level | School | 2.51 (2.61) | 0.929 ^b^ | 9.38 (5.73) | 0.618 ^b^ | 20.65 (13.10) | 0.737 ^b^ |
|  | Bachelor's | 2.49 (2.52) |  | 9.59 (5.95) |  | 21.47 (14.10) |  |
|  | Postgraduate | 2.64 (2.48) |  | 10.35 (5.88) |  | 22.35 (12.67) |  |
| Occupation Status | Unemployed | 2.57 (2.45) | ***0.002**** ^b^ | 8.71 (5.71) | 0.481 ^b^ | 20.61 (13.86) | 0.872 ^b^ |
|  | Employee | 2.47 (2.34) |  | 10.06 (5.79) |  | 22.14 (13.03) |  |
|  | Housewife | 1.96 (2.50) |  | 9.29 (5.99) |  | 20.68 (15.05) |  |
|  | Students | 3.37 (2.80) |  | 10.01 (6.18) |  | 21.89 (13.16) |  |
|  | Retired | 2.46 (2.47) |  | 9.73 (5.61) |  | 20.86 (12.96) |  |
| Income | <5000 SAR | 2.52 (2.74) | 0.100 ^b^ | 9.20 (6.09) | 0.273 ^b^ | 20.43 (14.07) | ***0.035**** ^b^ |
|  | 5000-10,000 SAR | 2.78 (2.33) |  | 10.15 (5.74) |  | 23.75 (13.37) |  |
|  | >10,000 SAR | 2.08 (2.24) |  | 9.81 (5.54) |  | 20.03 (12.89) |  |
| Smoking Status | No | 2.29 (2.55) | ***0.005**** ^b^ | 9.14 (5.94) | ***0.010**** ^b^ | 20.04 (13.84) | ***0.008**** ^b^ |
|  | Yes | 3.16 (2.30) |  | 9.97 (5.64) |  | 24.46 (13.89) |  |
|  | Passive Smoker | 3.15 (2.53) |  | 11.64 (5.52) |  | 25.00 (11.75) |  |
|  | Previous Smokers | 1.57 (1.51) |  | 9.14 (5.08) |  | 22.29 (15.33) |  |
| Chronic Diseases | No | 2.45 (2.50) | 0.623 ^a^ | 9.00 (6.30) | ***0.027**** ^a^ | 20.49 (14.34) | 0.178 ^a^ |
|  | Yes | 2.56 (2.57) |  | 10.16 (5.43) |  | 22.14 (13.06) |  |
| Doctor Visits | None | 3.10 (2.68) | ***0.009**** ^b^ | 9.17 (6.57) | 0.451 ^b^ | 21.03 (15.53) | 0.653 ^b^ |
|  | 1-4 Visits | 2.43 (2.48) |  | 9.93 (5.90) |  | 21.88 (13.57) |  |
|  | >5 Visits | 2.14 (2.43) |  | 9.37 (5.15) |  | 20.57 (12.14) |  |
| Gynecologic Surgery | No | 2.40 (2.48) | 0.373 ^a^ | 9.09 (5.64) | 0.090 ^a^ | 19.38 (12.53) | ***0.025**** ^a^ |
|  | Yes | 2.17 (2.50) |  | 10.10 (6.18) |  | 22.40 (14.38) |  |
| Mode of Delivery | Never | 2.80 (2.72) | ***0.026**** ^b^ | 8.82 (6.23) | 0.401 ^b^ | 18.84 (13.21) | 0. 145 ^b^ |
|  | Vaginal Delivery | 2.04 (2.27) |  | 9.87 (5.81) |  | 20.92 (13.25) |  |
|  | C-Section | 2.90 (3.04) |  | 9.55 (5.28) |  | 24.34 (13.57) |  |
|  | Both | 2.02 (2.32) |  | 10.15 (5.43) |  | 22.52 (13.45) |  |
| Menstrual History | Regular Period | 2.09 (2.39) | ***0.014**** ^b^ | 8.80 (6.08) | ***0.011**** ^b^ | 19.28 (14.08) | ***0.018**** ^b^ |
|  | Irregular Period | 2.94 (2.75) |  | 10.88 (5.59) |  | 23.85 (12.37) |  |
|  | Postmenopausal | 2.11 (2.32) |  | 9.97 (5.31) |  | 20.69 (11.74) |  |
| (a) Independent T-Test, (b) ANOVA, (*) Significant Values | | | | | | | |
